# The Impact of Psychiatric Care Program on Empathy of Neonatal Intensive Care Nurses in Iran

**DOI:** 10.4314/ejhs.v33i4.11

**Published:** 2023-07

**Authors:** Mohammad Heidarzadeh, Haydeh Heidari, Shima Heidary, Ali Ahmadi

**Affiliations:** 1 Neonatology, Faculty of Medicine, Zahedan University of Medical Sciences, Zahedan, Iran; 2 Associate professor, Modeling in Health Research Center, Shahrekord University of Medical Sciences, Shahrekord, Iran; 3 Faculty of Nursing and Midwifery, Shahrekord University of Medical Sciences, Shahrekord; 4 Associate Professor, Modeling in Health Research Center, Shahrekord University of Medical Sciences, Iran

**Keywords:** Empathy, Neonatal Intensive Care Unit, Education, Nurse

## Abstract

**Background:**

The main role of nurses is not only to inform about the disease and treatment of the patient but also to establish an effective therapeutic relationship to address concerns and provide empathy, comfort, and support. This issue is very prominent in neonatal intensive care units (NICUs) and doubles the importance of empathetic communication between nurses and parents and promoting empathy skills in nurses working in neonatal intensive care units.

**Aim:**

This study aimed to evaluate the effect of a support program on the empathy of neonatal intensive care nurses across the Iran.

**Methods:**

This study was conducted in 2021 as a semi-experimental intervention in a group of 166 nurses working in the neonatal department all over Iran who met the inclusion criteria. Jefferson's empathy questionnaire was completed electronically by the participants before and after the online education program start. Data were analyzed using SPSS software (V 24.0).

**Results:**

The empathy score of nurses was 60.31 ± 5.76 before and 66.47 ±6.60 after the intervention. The empathy scores of nurses after the intervention increased statistically significantly.

**Conclusion:**

Nurses can communicate empathically with parents by training their verbal and nonverbal communication skills and gaining a common understanding of the feelings of parents of premature infants.

## Introduction

Empathy is described as a cognitive and emotional attribute, or a combination of both, in nursing. Cognition in empathy is the mental activity, which involves acquiring and processing information for a better understanding (of the other, in this case). The emotional aspect is manifested by subjectively experiencing a feeling through empathy as an essential skill in social relationships in health. However, the emotional aspect is little studied in how it occurs in the context of professional nursing practices(1). The concept of empathy originates from the science of psychology and has integrated with other sciences such as medicine, psychiatry, and nursing. Empathy is perceiving others' feelings and views to effectively communicate with the premature infant-parent. Parental support by health care workers was considered a necessity. However, nurses do not consider parental support a primary concern because they think the workload is an obstacle for supporting parents in the special care ward (2).Empathy and empathic interaction are ways nurses can support parents of premature infants living in the NICU(3). However, few studies have analyzed how empathy happens to nurses working in the NICU. The importance of teaching empathetic communication and the role of empathy in nurse-parent communication should be more prominent (2-3).

The practice of nursing is infused with empathy, which improves and alters nurses' sensitivity to infants. Good clinical practice stresses that infant characteristics such as being away from parents do not draw attention because everyone is assisted. This aspect is present when empathy is how nurses perform their activities with babies. Despite the importance of empowering nurses with communication and empathy skills, the nursing curriculum should cover such organized skills. Nurses care for many patients per shift, especially those working in intensive care units. Due to particular circumstances, the patient in the intensive care unit requires multiple and 24-hour care, which may cause the nurse to miss the opportunity to communicate with the patient family (3-4).

Despite training on empathy in providing care, the results indicate low empathy skills among the healthcare team. A study of cancer patients found that oncologists responded with empathy to only 10 to 22% of patients' expressions of negative emotions. However, nurses rarely spend time talking and empathizing with patients (4). However, nurses rarely spend time talking and empathizing with patients. Inadequate training in communication skills, followed by poor empathy, exposes nurses and physicians to higher levels of emotional fatigue, job dissatisfaction, and increased healthcare conflicts (5-8).

In addition, the effectiveness of training has not been fully implemented by the nurses of the NICU ward (1). Emotional support of infants' parents in the NICU is one of the nurse's duties. Therefore, the treatment team needs training to promote empathic communication with the client. This study assessed a support program's effect on the NICU's empathy in Iran.

## Methods

This semi-experimental study was conducted in December 2021 on a single group of nurses working in the neonatal intensive care unit across Iran. The sample size was calculated based on Ghaedi's study (8)(Alpha=0.01, Power=0.95, M=87.51, SD=6.65, Ma=90), considering the possible drop rate of 10%, which increased to 200 nurses. A total of 166 nurses from 10 logistics regions and 42 provinces were selected to participate in the study through convenient sampling. The inclusion criteria were a licensed registered nurse, a permanent, casual, or contractual hospital nurse working more than three months, and consented to participate in the study. Furthermore, the exclusion criteria were a similar course at the same time, like holding a training workshop.

**Figure 1:**
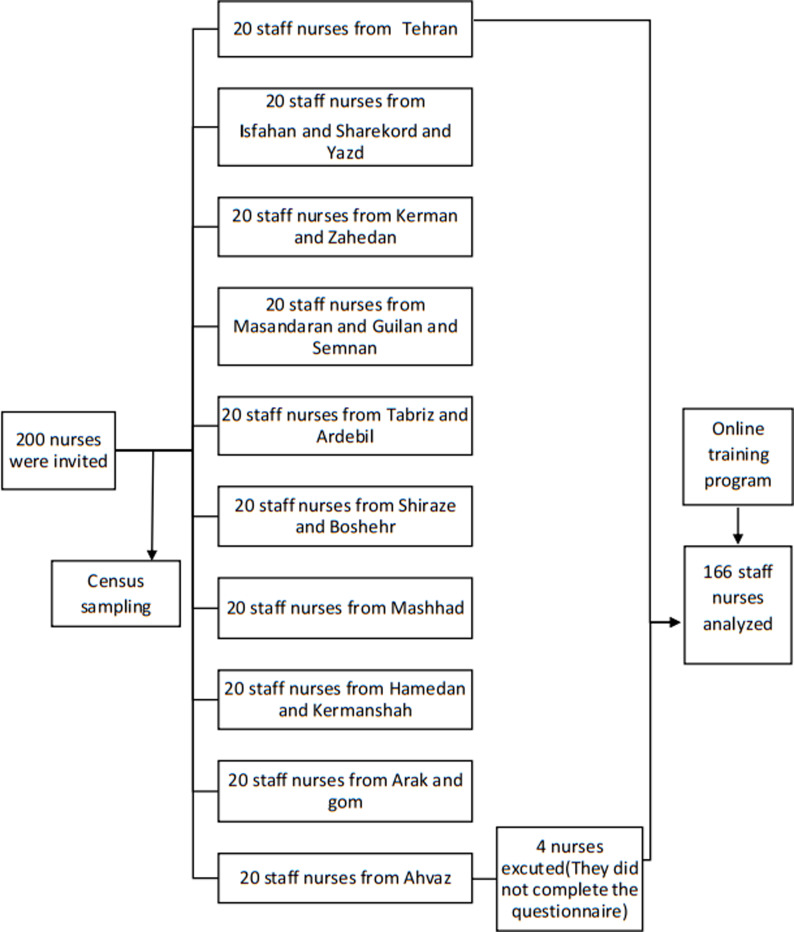
Flow chart study design

The samples were studied in an intervention group. Online sessions were held to assess the effectiveness of the care program (Figure 1). This study presented educational content for nurses based on Heidari et al. (Table 1).The educational content was revised using the Delphi method based on the opinions of eight experts (three physicians, three faculty members, and two health policymakers), and the final content was codified. Then, the educational content was finalized during two eight-hour sessions in Yazd, Iran, in the presence of experts. At the outset, the experts' opinions were applied to the initial principles during two sessions. Final results were presented to experts through a two-hour training program over six consecutive days (12 hours).

**Table 1:** Support letter and educational content

Training sessions	Support program in the neonatal intensive care unit	Training sessions	Training method
1. Holistic care and its importance in parental communication and the challenges of communication models	1-1-Difference in care and treatment, 2-1-How to assess the needs of parents 3-1-Types of communication models	1. Holistic care and its importance in parental communication and the challenges of communication models	Lectures, presentation of scenarios, questions, and answers
2. Communication with parents in the neonatal intensive care unit	1-2-Empathy 2-2- Reassurance 3-2- Comfort 4-2- Dignity and respect 5-2-Awareness of parents' needs	2. Communication with parents in the neonatal intensive care unit	Lectures, presentation of scenarios, questions, and answers
3- Special measures to communicate with families in the neonatal visa care department	1-3-Before meeting 2-3-During the meeting 3-3-Follow-up after the meeting	3- Special measures to communicate with families in the neonatal visa care department	Lectures, presentation of scenarios, questions, and answers
4. Principles of infant and family care	1-4-Decision: 2-4-Family adjustment: 3-4- Personnel stress in relation to family interaction	4. Principles of infant and family care	Lectures, presentation of scenarios, questions, and answers
5- Paying attention to the religion and culture of the family	4-4-Family cultural support 5-4-Religious support	5- Paying attention to the religion and culture of the family	Lectures, presentation of scenarios, questions, and answers
6- How parents participate Training sessions	6-4-Family visit 7-4- Family presence in the round 8-4- Presence of parents in resuscitation	6- How parents participate	Lectures, presentation of scenarios, questions, and answers

Participants completed the Jefferson Scale of Empathy (JSE) (Health Professions) in the pre-test before the electronic start of the program and in the post-test after the intervention. Based on the agreement rate, the completer scored each item from one to five (one indicates complete disagreement and five shows the entire agreement). The minimum score is 20, and the maximum is 100. The validity and reliability of the scale were 83%, and the data were analyzed using SPSS software version 24 and paired t-tests.

**Ethics and Other Permissions**: The present article with the ethics code IR.SKUMS.REC.1400.230 is from the ethics committee of the School of Nursing and Midwifery of Shahrekord University of Medical Sciences.

## Results

The results showed that out of the 166 nurses, 65.1% were female (108 People), and 34.9% were male (58 People). About 24.7% of the people (41 People) had less than five years of experience, 36.7% (61 People) had 5-10 years, 19.9% (33 People) had 10-20 years, and 18.7% (31 People) had 20-30 years of work experience. A total of 9% (15 People) of people were in the range of 20-30 years of age, 12%(20 People) were in the range of 30-40 years, 30% (50 People) were in the range of 40-50 years, and 48.8% (81 People) were over 50 years of age. The post-test empathy score differed significantly from the pre-test, proving the hypothesis. Empowering nurses increased their empathy in the NICU.

## Discussion

Nurses' empathetic responses were significant in an acute clinical setting like the neonatal intensive care unit(12). Empathy in patient care was a competence beyond mere clinical skills, emphasizing communication and ethics(13). Developing effective training programs created the required qualifications for a nurse or physician. The present study showed that a supportive training program is effective in nurses' empathy and has increased their empathy for the families of premature infants(14).Some studies have indicated that the communication between nurses and patients is weak, and nurses rarely spend time talking and empathizing with patients. Another study found that nurses' empathy level in the intensive care unit (ICU) was moderate. However, another study revealed that the empathy scores of nurses working in the ICU were the lowest. Stavropoulou et al. stated that nurses emphasized that understaffing, increased workload, and burnout impeded compassionate care. Empathy and compassionate care in the ICU are closely related to patient outcomes and quality of care. In practice, critical care nurses aim to strengthen compassionate care (15, 16).

Empathy training enhances the nurses' empathy and social and psychological competence. Increasing empathy may enable nurses to eliminate negative emotions such as anxiety, depression, and irritability. Empathy training for ICU nurses who interact most with patients and their families can meet the psychological needs of the patients and their companions. Nurses learn to know their patients, just as a mother learns to interpret their children's crying reason through experience. An infant's expression interpreted by nurses would indirectly give them the object of their experience. Nurses face more challenging situations and responsibilities in some wards, including emergency, psychiatry, ICU, and NICU, than other nurses. Communication with patients is difficult for nurses in stressful work environments, and they do not have enough opportunities to deal with patients. However, there is limited information on the level of empathy of nurses in the neonatal intensive care unit(17, 18). Some reasons for poor levels of empathy may include workload, time constraints, lack of education, and ignoring the role of empathy in family-centered care, causing less attention to the human dimension in patient care (19).

The communication technique courses improve nurses' ability to empathize with parental feelings, and the nurses felt that these courses benefited their profession. As expected, most conversations focused on illness and infant care, and the nurses discussed psychological and social issues. In addition, applications such as hospital rules and programs in other fields, such as psychological aspects of social issues, such as family life outside the hospital, were rarely discussed(12). Nurses and parents can improve communication to increase parental involvement and make infant care more family-oriented(20).

In a qualitative study, Babaii et al. stated that the empathic relationship is appropriate to the conditions and needs of hospitalized patients. Listening to parents of premature babies, providing information, and encouraging them to express their concerns is important for maintaining a close relationship with them (21).The family's needs can be met by increasing the empathy skills of the nurses, and nurses can encourage family members to participate in planning their baby's care through empathic communication. These studies showed that different educational methods, such as practical exercises, scenario statements, discussions, role-playing games, assignments, question-and-answer sessions, and lectures, improve nurses' and doctors' empathy skills (22-24).

Experimental learning can improve learners' empathy by integrating practical learning and increasing the quality of care for future health professionals. The empathy score of nursing students in managing violent patients improved after participating in an experimental learning session(25). Although the importance of empathy is undeniable, many health professionals seem to have difficulty adopting a model of empathetic communication in their daily activities(26). Similar to the present study and based on parental feedback, the workshop's learning objectives were to provide medical information to families with better communication and empathy skills. Another objective was to improve communication skills in telling “bad news,” understanding nonverbal communication between employees and family, and learning skills to help manage families(27). Mirzaei et al. concluded that empathy is necessary for effective nursing care. The present intervention showed the effectiveness of empathy training on individuals. Nurses' empathy skills can be acquired or learned, and an empathetic relationship between nurse and patient leads to positive treatment outcomes (16).

Although nursing in the intensive care unit is stressful and faces serious challenges, feeling frustrated by failures and relying on personal abilities produces positive benefits and results in nurses' empathetic abilities. Nurses' performance is very influential in advancing organizational goals, and responsibility and appropriate patient treatment play a vital role in fulfilling the health system's mission. Nurses must first be able to communicate well with the patient to provide high-quality care (28, 29).

In conclusion, the medical staff, especially nurses, should take extra care of patients' families in the intensive care unit. Increasing the level of empathy of nurses with premature family members can play an influential role in meeting the patients' parents' needs in the special care wards. Based on the studies, compassionate care is a deep interaction between nurses and doctors with families. Healthcare workers can communicate with the necessary care by strengthening their verbal and non-verbal communication skills and using empathy. Understanding the feelings of parents of premature babies can help explore the many pains of families of premature babies because empathy skills can be learned, taught, and improved. These findings suggest that hospitals should conduct in-service training programs to strengthen the empathy of nurses and doctors while providing regular health care to the treatment team. Managers of healthcare organizations can take practical steps to implement compassionate care programs using the facilities of the organization, persuading and encouraging caring nurses, utilizing the individual capacities of nurses, and creating specialized teams. Therefore, paying attention to the empathy skills of nurses working in ICUs increases the quality and quantity of their provided healthcare. Moreover, training can greatly help nurses' empathy, and nurses can be more useful caregivers in the healthcare system if trained effectively, making them more satisfied and relaxed.

## Figures and Tables

**Table 2:** Comparison of mean empathy score before and after the intervention

Test time	Mean±standard deviation	Total
Pre-test	60/31 ± 5/76	6/15 ± 6/8
Post-test	66/47 ± 6/60	T=11/65 , ***DF^#^***=165 , p* < 0/0001

Notes: ***DF^#^***= Degrees of freedom, p*=P value

## References

[1] Mufato LF, Gaíva MA (2022). Nurses' empathy with newborns hospitalized in neonatal intensive care units. Acta Paul. de Enferm.

[2] Negarandeh R, Hassankhani H, Jabraeili M, Abbaszadeh M, Best A (2021). Health care staff support for mothers in NICU: a focused ethnography study. BMC Pregnancy Childbirth.

[3] Mohagheghi P, Keramat A, Chaman R, Khosravi A, Mousavi S, Mousavi S (2020). Effect of Early Support on the Stress of Mothers with Preterm Infants in Neonatal Intensive Care Units: A Quasi-experimental Study. IJN.

[4] Kesbakhi MS, Rohani C, Mohtashami J, Nasiri M (2017). Empathy from the perspective of oncology nurses. J. Compassionate Health Care.

[5] Eby D (2018). Empathy in general practice: its meaning for patients and doctors. British Journal of General Practice.

[6] Kerasidou A, Bærøe K, Berger Z, Brown AEC (2021). The need for empathetic healthcare systems. J. Med. Ethics.

[7] Bas-Sarmiento P, Fernández-Gutiérrez M, Baena-Baños M, Correro-Bermejo A, Soler-Martins PS, de la Torre-Moyano S (2020). Empathy training in health sciences: A systematic review. J Nurs Educ. Pract.

[8] Ghaedi F, Ashouri E, Soheili M, Sahragerd M (2020). Nurses' empathy in different wards: A cross-sectional study. INJMR.

[9] Heidari H, Heidarzadeh M (2017). Developing the principles of parental mental health in the Neonatal Intensive Care Unit (NICU). IJN.

[10] Rahmani H, Maleki R, Ghanbari MK, Behzadifar M (2022). Quality Assessment of Services in Primary Healthcare in Iran: A Systematic Review and Meta-analysis. Ethiop. J. Health Sci.

[11] Ezzati R, Tafazoli M, Mazlom SR (2018). Assessment of Empathy Skills in Midwifery Students and its Relationship with Some of the Demographic Factors. Nurs midwifery res. j.

[12] Ayuso-Murillo D, Colomer-Sánchez A, Santiago-Magdalena CR, Lendínez-Mesa A, Gracia EBD, López-Peláez A (2020). Effect of Anxiety on Empathy: An Observational Study Among Nurses. Healthcare (Basel).

[13] Avci D, Alp Yilmaz F (2021). Association between Turkish clinical nurses' perceptions of individualized care and empathic tendencies. Perspect. Psychiatr. Care.

[14] Zeraatchi A, Zeraati M, Samieerad F, Rostami M (2021). Needs Assessment of Continuing Education Programs for Nursing Staff Working in Zanjan University of Medical Sciences. Research in Medical Education.

[15] Stavropoulou A, Rovithis M, Sigala E, Pantou S, Koukouli S (2020). Greek nurses' perceptions on empathy and empathic care in the Intensive Care Unit. Intensive Crit. Care Nurs.

[16] Mirzaei Maghsud A, Abazari F, Miri S, Sadat Nematollahi M (2020). The effectiveness of empathy training on the empathy skills of nurses working in intensive care units. J Res Nurs.

[17] Zamani P, Dehnad A, Haghani H, Borimnejad L (2019). Effect of Web-Based Education on Knowledge, Attitude, and Practice of Nurses in Neonatal Intensive Care Unit. Interdiscip. j. virtual learn. med. sci.

[18] Hall SL, Famuyide ME, Saxton SN, Moore TA, Mosher S, Sorrells K (2019). Improving staff knowledge and attitudes toward providing psychosocial support to NICU parents through an online education course. Adv. Neonatal Care.

[19] Winter R, Issa E, Roberts N, Norman RI, Howick J (2020). Assessing the effect of empathy-enhancing interventions in health education and training: a systematic review of randomised controlled trials. BMJ open.

[20] Bry K, Bry M, Hentz E, Karlsson HL, Kyllönen H, Lundkvist M (2016). Communication skills training enhances nurses' ability to respond with empathy to parents' emotions in a neonatal intensive care unit. Acta paediatr.

[21] Babaii A, Mohammadi E, Sadooghiasl A (2021). The Meaning of the Empathetic Nurse–Patient Communication: A Qualitative Study. J. Patient Exp.

[22] Kahriman I, Nural N, Arslan U, Topbas M, Can G, Kasim S (2016). The effect of empathy training on the empathic skills of nurses. Iran. Red. Crescent. Med.

[23] Gholamzadeh S, Khastavaneh M, Khademian Z, Ghadakpour S (2018). The effects of empathy skills training on nursing students' empathy and attitudes toward elderly people. BMC Med Educ.

[24] Guven Ozdemir N, Sendir M (2020). The relationship between nurses' empathic tendencies, empathic skills, and individualized care perceptions. Perspect. Psychiatr. Care.

[25] Goh Y-S, Seetoh Y-TM, Chng M-L, Ong SL, Li Z, Hu Y (2020). Using Empathetic CAre and REsponse (ECARE) in improving empathy and confidence among nursing and medical students when managing dangerous, aggressive and violent patients in the clinical setting. Nurse Educ Today.

[26] Moudatsou M, Stavropoulou A, Philalithis A, Koukouli S (2020). The role of empathy in health and social care professionals. Healthcare.

[27] Kasat K, Stoffels G, Ellington M (2020). Improving communication with parents: the Neonatal Intensive Care Unit Empathy Workshop. J Perinatol.

[28] Heidari H, Hasanpour M, Fooladi M, Feizi A (2015). Construction of a questionnaire to assess parental stress in neonatal intensive care unit. IJN.

[29] Mahdian M, Sadeghi N, Mohammady M (2021). The effects of empathy techniques education for nurses on resilience and spiritual well-being among mothers with preterm neonates in neonatal intensive care unit. J Multidiscip Care.

